# Tobacco Control in Cancer Care

**DOI:** 10.1097/JNR.0000000000000353

**Published:** 2019-09-20

**Authors:** Shu-Ching CHEN

Tobacco smoking has been directly associated with many types of cancer. Six million people die from tobacco smoking globally each year (World Health Organization, 2019), and according to the Centers for Disease Control and Prevention's Division of Cancer Prevention and Control, more than 600,000 patients are diagnosed with and around 350,000 patients die from tobacco-related cancers in the United States each year (Centers for Disease Control and Prevention, 2019). In Taiwan, an estimated 18.0% of adults smoke tobacco, which is known to negatively affect human health and to be a risk factor for cancer (Health Promotion Administration, Ministry of Health and Welfare, ROC, 2018).

Controlling tobacco consumption reduces cancer risk, promotes early cancer detection, increases treatment efficacy, improves treatment-related toxicity, controls disease progression, prevents cancer recurrence, reduces the risk of secondary cancers, and increases survival and quality of life (Gajdos et al., 2012). Although healthcare providers implement tobacco control measures in oncology settings, a national survey by a cancer institute in the United States revealed that only 62% of cancer institutes provided tobacco educational materials routinely to patients. Moreover, over half of those that routinely provided these materials maintained an effective identification system to detect patient tobacco use, with the remaining institutes either having an employee on staff who was dedicated to providing tobacco use reduction services or having an executive team that was committed to providing tobacco use treatment services (Goldstein, Ripley-Moffitt, Pathman, & Patsakham, 2013). Tobacco control is a considerable challenge in cancer care, and the lack of appropriate tobacco control measures may limit the effectiveness of this care.

In this issue of The Journal of Nursing Research, Kurt and Akyuz evaluate whether educational brochure intervention increased the number of women who were screened for cervical cancer. The brochure-and-education group attained higher knowledge scores and a higher cervical cancer screening rate than the other two groups. This finding may encourage healthcare providers to provide cancer preventive care education and materials to patients and facilitate the early detection of cancers. Future studies should identify the smoking status and tobacco-control-related information that is most useful for inclusion in these educational brochures. In addition, Tsai et al. analyzed the Assessment of Survivor Concerns scale for gynecological cancer survivors, revealing that cancer survivors are concerned about secondary cancers, cancer recurrence, and overall health status. Thus, tobacco control is crucial to attaining effective survivorship care and increasing patient survival and quality of life.

## References

[bib1] Centers for Disease Control and Prevention. (2019). *Classification of diseases, functioning, and disability*. Retrieved from http://www.cdc.gov/media/releases/2016/p1110-vital-signs-tobaccohtml.206.null

[bib2] Gajdos C. Hawn M. Campagna E. Henderson W. Singh J. & Houston T. (2012). Adverse effects of smoking on postoperative outcomes in cancer patients. *Annals of Surgical Oncology*, 19(5), 1430–1438. 10.1245/s10434-011-2128-y 22065194 PMC4262530

[bib3] Goldstein A. O. Ripley-Moffitt C. E. Pathman D. E. & Patsakham K. M. (2013). Tobacco use treatment at the U.S. national cancer institute's designated cancer centers. *Nicotine and Tobacco Research*, 15(1), 52–58. 10.1093/ntr/nts083 22499079 PMC3842130

[bib4] Health Promotion Administration, Ministry of Health and Welfare, ROC. (2018). *Taiwan tobacco control annual report 2018*. Retrieved from http://www.hpa.gov.tw

[bib5] World Health Organization. (2019). *Fact sheet on tobacco*. Retrieved from http://www.who.int/mediacentre/factsheets/fs339/en/

